# Supplementary material for: The social network of medical case managers, housing providers, and health department staff in the Ryan White HIV/AIDS Program: A Midwest case study
Source: PLoS One. 2020 Aug 28;15(8):e0238430. doi: 10.1371/journal.pone.0238430 (PMC7454952; doi:10.1371/journal.pone.0238430)
Supplement: S3 File — (DOCX) [file pone.0238430.s003.docx]

Service Coordination for HIV Medical Case Management and Housing Support Services

Survey Flow

Standard: Block 3 (1 Question)

Standard: Block 1 (28 Questions)

Standard: Housing and Health Outcomes Report (7 Questions)

Standard: Housing Referral Checklist (5 Questions)

Standard: Social Network Analysis (4 Questions)

Block: Default Question Block (6 Questions)

Standard: Block 6 (1 Question)

| Page Break |  |
| --- | --- |

Start of Block: Block 3

Thank you for participating in the following survey about service coordination among Medical Case Managers and Housing Providers in the Kansas City. The following questions will ask you about SCOUT and generally how you work together between and across agencies. This survey should take approximately 15-20 minutes to complete.  There are no known risks of participating in this survey. Your participation is completely voluntary. You may stop or refuse to answer any question at any time simply by closing your browser window. All data are confidential. Only Dr. Joey Lightner will have access to your individual responses. If you have any questions, please contact Dr. Joey Lightner lightnerj@umkc.edu before you begin. By clicking "next" you agree to participate. Your participation in this research is voluntary, and you will not be penalized if you refuse to participate or stop.

End of Block: Block 3

Start of Block: Block 1

Please type your first and last name

________________________________________________________________

| 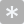 |
| --- |

What is your age in years?

________________________________________________________________

What is your gender (Click one)?

- Male
- Female
- Male-to-Female or Female-to-Male Transgender
- other

How would you describe your race and ethnicity (Click all that apply)?

- American Indian or Alaska Native
- Asian
- Black or African American
- Latino
- Native Hawaiian or other Pacific Islander
- White
- Other

What is your highest level of education that you have completed (Click one)?

- Less than high school diploma
- High school diploma or GED
- Some college, but no degree
- College degree
- Some graduate school, but no degree
- Graduate degree

Do you provide direct services to Ryan White and/or HOPWA clients?

- Yes, to Ryan White clients
- Yes, to HOPWA clients
- Yes, to both Ryan White and HOPWA clients
- No, I do not provide direct services

Are you a Housing Provider or Medical Case Manager?

- Housing Provider
- Medical Case Manager

| Page Break |  |
| --- | --- |

What is your current job title?

________________________________________________________________

| 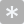 |
| --- |

How long have you held this position (in years)?

________________________________________________________________

What are your primary job responsibilities?

________________________________________________________________

Display This Question:

If Are you a Housing Provider or Medical Case Manager? = Housing Provider

When you respond to the next items, please think about SCOUT including the Housing and Health Outcomes Report, where you are able to view medical information.

Display This Question:

If Are you a Housing Provider or Medical Case Manager? = Housing Provider

In the past 6 months, to what extent have you used SCOUT to view medical information on your clients?

- Not at all
- Rarely (less than once a month)
- Sometimes (about once a month)
- Often (about 1-2 times per week)
- Very Often (every day or almost every day)

Skip To: Q21 If In the past 6 months, to what extent have you used SCOUT to view medical information on your clie... = Often (about 1-2 times per week)

Skip To: Q21 If In the past 6 months, to what extent have you used SCOUT to view medical information on your clie... = Very Often (every day or almost every day)

Display This Question:

If Are you a Housing Provider or Medical Case Manager? = Housing Provider

Which of the following are reasons why you do not use SCOUT to view medical information on your clients often?

- NA - I use SCOUT for all or most of my clients
- I was not told to use SCOUT
- I did not receive adequate training in SCOUT
- I think that SCOUT is hard to use
- I do not think that I need SCOUT to provide client services
- Other

Display This Question:

If Are you a Housing Provider or Medical Case Manager? = Housing Provider

In the past 6 months, how have you used SCOUT? (Click all that apply)

- I have not used SCOUT
- To check client health indicators (e.g. viral load)
- To upload electronic documents that can be viewed in SCOUT
- To label the electronic documents that can be viewed in SCOUT
- To communicate with other providers about clients
- To list myself as a professional contact
- Other

Display This Question:

If Are you a Housing Provider or Medical Case Manager? = Housing Provider

In the past 6 months, to what extent do you think that other providers in your organization use SCOUT to view medical information about clients? Your best estimate is fine.

- Not at all
- Rarely (less than once a month)
- Sometimes (about once a month)
- Often (about 1-2 times per week)
- Very Often (every day or almost every day)

Display This Question:

If Are you a Housing Provider or Medical Case Manager? = Housing Provider

| 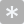 |
| --- |

In the past 6 months, approximately how many hours of training did you receive on SCOUT (in hours)?

________________________________________________________________

Display This Question:

If Are you a Housing Provider or Medical Case Manager? = Housing Provider

To what extent do you agree or disagree with the following statement? <div><br></div><div>"I feel that I have had enough training in SCOUT to use it correctly." <div><br></div></div>

- Strongly Disagree
- Slightly Disagree
- Neutral
- Slightly Agree
- Strongly Agree

Display This Question:

If Are you a Housing Provider or Medical Case Manager? = Housing Provider

What do you think are the most challenging aspect of using SCOUT?

- Most challenging ________________________________________________
- Second most challenging ________________________________________________
- Third most challenging ________________________________________________

Display This Question:

If Are you a Housing Provider or Medical Case Manager? = Housing Provider

What do you think are the most positive aspects of using SCOUT?

- Most positive ________________________________________________
- Second most positive ________________________________________________
- Third most positive ________________________________________________

Display This Question:

If Are you a Housing Provider or Medical Case Manager? = Medical Case Manager

Next, we'd like you to think about your interaction with SCOUT, including for the purposes of medical case management and housing services.

Display This Question:

If Are you a Housing Provider or Medical Case Manager? = Medical Case Manager

In  the past 6 months, to what extent have you used SCOUT regarding housing information about clients?

- Not at all
- Rarely (less than once a month)
- Sometimes (about once a month)
- Often (about 1-2 times per week)
- Very Often (every day or almost every day)

Skip To: Q49 If In the past 6 months, to what extent have you used SCOUT regarding housing information about clie... = Often (about 1-2 times per week)

Skip To: Q49 If In the past 6 months, to what extent have you used SCOUT regarding housing information about clie... = Very Often (every day or almost every day)

Display This Question:

If Are you a Housing Provider or Medical Case Manager? = Medical Case Manager

Which of the following are reasons why you do not access housing information in SCOUT often?

- N/A -- I use SCOUT for all or most of my clients
- I was not told to use SCOUT
- I did not receive adequate training in SCOUT
- I think that SCOUT is hard to use
- I do not think that I need SCOUT to provide client services
- Other

Display This Question:

If Are you a Housing Provider or Medical Case Manager? = Medical Case Manager

In the past 6 months, to what extent do you think that other medical case managers in your organization used SCOUT regarding housing information about clients? Your best estimate is fine.

- Not at all
- Rarely (less than once a month)
- Sometimes (about once a month)
- Often (about 1-2 times per week)
- Very Often (every day or almost every day)

Display This Question:

If Are you a Housing Provider or Medical Case Manager? = Medical Case Manager

In the past 6 months, how have you used housing components in SCOUT? (Check all that apply)

- N/A -- I have not used SCOUT
- To use the "KC Housing Voucher Lottery ENT" workflow
- To view documents made electronically available by Housing Providers
- To identify a client's Housing Specialist
- To communicate with other providers about clients
- Other

Display This Question:

If Are you a Housing Provider or Medical Case Manager? = Medical Case Manager

| 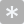 |
| --- |

In the past 6 months, approximately how many hours of training did you receive on using the new housing components in SCOUT? (in hours)

________________________________________________________________

Display This Question:

If Are you a Housing Provider or Medical Case Manager? = Medical Case Manager

To what extent do you agree or disagree with the following statement? "I feel that I have had enough training on the new housing components in SCOUT to use them correctly."

- Strongly Disagree
- Disagree
- Neutral
- Agree
- Strongly agree

Display This Question:

If Are you a Housing Provider or Medical Case Manager? = Medical Case Manager

What do you think are the most challenging aspects of using the new housing components in SCOUT?

- Most challenging ________________________________________________
- Second most challenging ________________________________________________
- Third most challenging ________________________________________________

Display This Question:

If Are you a Housing Provider or Medical Case Manager? = Medical Case Manager

What do you think are the most positive aspects of using the new housing components in SCOUT?

- Most positive ________________________________________________
- Second most positive ________________________________________________
- Third most positive ________________________________________________

End of Block: Block 1

Start of Block: Housing and Health Outcomes Report

Display This Question:

If Are you a Housing Provider or Medical Case Manager? = Housing Provider

The following questions will assess the use of the Housing and Health Outcomes Report. When answering these questions please think about the Housing and Health Outcomes Report.

Display This Question:

If Are you a Housing Provider or Medical Case Manager? = Housing Provider

|  | Never used the report | Never | Once | Multiple times | Everyday |
| --- | --- | --- | --- | --- | --- |
| How often have you used the Housing and Health Outcomes Report? |  |  |  |  |  |
| How often has your supervisor requested you use the Housing and Health Outcomes Report? |  |  |  |  |  |

Display This Question:

If Are you a Housing Provider or Medical Case Manager? = Housing Provider

|  | Never used the report | Not at all | Somewhat | Very |
| --- | --- | --- | --- | --- |
| How valuable do you feel the Housing and Health Outcomes Report is for your job? |  |  |  |  |
| How comfortable do you feel running the report? |  |  |  |  |
| How comfortable are you interpreting the results of the report? |  |  |  |  |

Display This Question:

If Are you a Housing Provider or Medical Case Manager? = Housing Provider

If you have used the Housing and Health Outcomes Report, please select why (Click all that apply).

- To see if client is virally suppressed
- To see if client has been to the doctor in the past 6 months
- To see if client has AIDS Drug Assistance Program (ADAP)
- To discuss current health concerns with client
- To know my caseload's percent VLS
- To coordinate with medical case managers
- To improve my understanding of my caseload's health status

Display This Question:

If Are you a Housing Provider or Medical Case Manager? = Housing Provider

What prompts you to run the Housing and Health Outcomes Report (Click all that apply)?

- I don't use the Housing and Health Outcomes Report
- It is routine
- I run it when I have a meeting with a client
- My supervisor requests that I use it
- I use it to manage my time

Display This Question:

If Are you a Housing Provider or Medical Case Manager? = Housing Provider

If you have used the Housing and Health Outcomes Report to talk with clients, please describe how it affected your conversations with clients, if at all.

________________________________________________________________

Display This Question:

If Are you a Housing Provider or Medical Case Manager? = Housing Provider

If you could improve the Housing and Health Outcomes Report, how would you improve it?

________________________________________________________________

End of Block: Housing and Health Outcomes Report

Start of Block: Housing Referral Checklist

Display This Question:

If Are you a Housing Provider or Medical Case Manager? = Medical Case Manager

The following questions will ask you about the Housing Referral Checklist.

Display This Question:

If Are you a Housing Provider or Medical Case Manager? = Medical Case Manager

Have you used the Housing Referral Checklist?

- Yes
- No

Display This Question:

If Are you a Housing Provider or Medical Case Manager? = Medical Case Manager

How often have you used the Housing Referral Checklist?

- Never
- Rarely
- Sometimes
- Often
- Everyday

Display This Question:

If Are you a Housing Provider or Medical Case Manager? = Medical Case Manager

How useful is the Housing Referral Checklist?

- Not useful
- A little useful
- Somewhat useful
- Useful
- Very useful

Display This Question:

If Are you a Housing Provider or Medical Case Manager? = Medical Case Manager

If you could improve the Housing Referral Checklist, how would you improve it?

________________________________________________________________

End of Block: Housing Referral Checklist

Start of Block: Social Network Analysis

The following will assess other things associated with service coordination.

|  | Not comfortable | Somewhat comfortable | Comfortable | Very Comfortable |
| --- | --- | --- | --- | --- |
| How comfortable do you feel discussing health matters with your clients? |  |  |  |  |
| Overall, how comfortable are you bringing up the topic of health the your clients? |  |  |  |  |
| How comfortable are you initiating conversations about health with your clients? |  |  |  |  |
| How comfortable are you discussing housing matter with your clients? |  |  |  |  |

Are there specific topics around housing that you are uncomfortable discussing with clients? If so, what are they?

________________________________________________________________

Are there specific health issues that you are uncomfortable discussing with your clients?

________________________________________________________________

End of Block: Social Network Analysis

Start of Block: Default Question Block

Please<span class="MsoCommentReference"><span style="font-size: 8pt; line-height: 11.4133px;"> </span></span>identify 10 people who are important to you in your professional network around housing and healthcare. These can be people who provide you with information to do your work, help you think about complex problems posed by housing and healthcare, or provide advice in your day-to-day life around this project. These individuals can be at your organization or any other organization.

|  | Where do they work? | What is their relationship to you (e.g. colleague, supervisor, subordinate, etc)? |
| --- | --- | --- |
| 1 |  |  |
| 2 |  |  |
| 3 |  |  |
| 4 |  |  |
| 5 |  |  |
| 6 |  |  |
| 7 |  |  |
| 8 |  |  |
| 9 |  |  |
| 10 |  |  |

| Page Break |  |
| --- | --- |

Please identify the primary benefit you receive from them in your work life (Please click one).

|  | Help me to understand health issues | Help me to understand housing issues | Help me navigate the housing referral process | Improve my comfort level when talking about health with consumers | Is a friend I can talk to about consumer issues | Is a friend I can talk to about navigating the medical case management process | Other |
| --- | --- | --- | --- | --- | --- | --- | --- |
| ${q://QID2/ChoiceTextEntryValue/1} |  |  |  |  |  |  |  |
| ${q://QID2/ChoiceTextEntryValue/2} |  |  |  |  |  |  |  |
| ${q://QID2/ChoiceTextEntryValue/3} |  |  |  |  |  |  |  |
| ${q://QID2/ChoiceTextEntryValue/4} |  |  |  |  |  |  |  |
| ${q://QID2/ChoiceTextEntryValue/5} |  |  |  |  |  |  |  |
| ${q://QID2/ChoiceTextEntryValue/6} |  |  |  |  |  |  |  |
| ${q://QID2/ChoiceTextEntryValue/7} |  |  |  |  |  |  |  |
| ${q://QID2/ChoiceTextEntryValue/8} |  |  |  |  |  |  |  |
| ${q://QID2/ChoiceTextEntryValue/9} |  |  |  |  |  |  |  |
| ${q://QID2/ChoiceTextEntryValue/10} |  |  |  |  |  |  |  |

For each person please indicate how often you interact with them.

|  | Rarely (almost never) | Occasionally (monthly) | Frequently (weekly) | Very Frequently (daily) |
| --- | --- | --- | --- | --- |
| ${q://QID2/ChoiceTextEntryValue/1} |  |  |  |  |
| ${q://QID2/ChoiceTextEntryValue/2} |  |  |  |  |
| ${q://QID2/ChoiceTextEntryValue/3} |  |  |  |  |
| ${q://QID2/ChoiceTextEntryValue/4} |  |  |  |  |
| ${q://QID2/ChoiceTextEntryValue/5} |  |  |  |  |
| ${q://QID2/ChoiceTextEntryValue/6} |  |  |  |  |
| ${q://QID2/ChoiceTextEntryValue/7} |  |  |  |  |
| ${q://QID2/ChoiceTextEntryValue/8} |  |  |  |  |
| ${q://QID2/ChoiceTextEntryValue/9} |  |  |  |  |
| ${q://QID2/ChoiceTextEntryValue/10} |  |  |  |  |

For each person please indicate how much you value their input when doing your job.

|  | Not at all valuable | Somewhat valuable | Valuable | Very Valuable |
| --- | --- | --- | --- | --- |
| ${q://QID2/ChoiceTextEntryValue/1} |  |  |  |  |
| ${q://QID2/ChoiceTextEntryValue/2} |  |  |  |  |
| ${q://QID2/ChoiceTextEntryValue/3} |  |  |  |  |
| ${q://QID2/ChoiceTextEntryValue/4} |  |  |  |  |
| ${q://QID2/ChoiceTextEntryValue/5} |  |  |  |  |
| ${q://QID2/ChoiceTextEntryValue/6} |  |  |  |  |
| ${q://QID2/ChoiceTextEntryValue/7} |  |  |  |  |
| ${q://QID2/ChoiceTextEntryValue/8} |  |  |  |  |
| ${q://QID2/ChoiceTextEntryValue/9} |  |  |  |  |
| ${q://QID2/ChoiceTextEntryValue/10} |  |  |  |  |

| 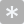 |
| --- |

How long have you know each person? Please provide answers in years (e.g. a year and a half is 1.5 years)

|  | Years |
| --- | --- |
| ${q://QID2/ChoiceTextEntryValue/1} |  |
| ${q://QID2/ChoiceTextEntryValue/2} |  |
| ${q://QID2/ChoiceTextEntryValue/3} |  |
| ${q://QID2/ChoiceTextEntryValue/4} |  |
| ${q://QID2/ChoiceTextEntryValue/5} |  |
| ${q://QID2/ChoiceTextEntryValue/6} |  |
| ${q://QID2/ChoiceTextEntryValue/7} |  |
| ${q://QID2/ChoiceTextEntryValue/8} |  |
| ${q://QID2/ChoiceTextEntryValue/9} |  |
| ${q://QID2/ChoiceTextEntryValue/10} |  |

How well do you know each person?

|  | Extremely well | Very well | Moderately well | Slightly well | Not well at all |
| --- | --- | --- | --- | --- | --- |
| ${q://QID2/ChoiceTextEntryValue/1} |  |  |  |  |  |
| ${q://QID2/ChoiceTextEntryValue/2} |  |  |  |  |  |
| ${q://QID2/ChoiceTextEntryValue/3} |  |  |  |  |  |
| ${q://QID2/ChoiceTextEntryValue/4} |  |  |  |  |  |
| ${q://QID2/ChoiceTextEntryValue/5} |  |  |  |  |  |
| ${q://QID2/ChoiceTextEntryValue/6} |  |  |  |  |  |
| ${q://QID2/ChoiceTextEntryValue/7} |  |  |  |  |  |
| ${q://QID2/ChoiceTextEntryValue/8} |  |  |  |  |  |
| ${q://QID2/ChoiceTextEntryValue/9} |  |  |  |  |  |
| ${q://QID2/ChoiceTextEntryValue/10} |  |  |  |  |  |

End of Block: Default Question Block

Start of Block: Block 6

Thank you for your thoughtful responses. Please click <a href="https://umkc.co1.qualtrics.com/jfe/form/SV_0ob9PVzETrm4l7f">HERE</a> to submit your email address and receive your $5 Amazon gift card!

End of Block: Block 6
