# Supplementary material for: The social network of medical case managers, housing providers, and health department staff in the Ryan White HIV/AIDS Program: A Midwest case study
Source: PLoS One. 2020 Aug 28;15(8):e0238430. doi: 10.1371/journal.pone.0238430 (PMC7454952; doi:10.1371/journal.pone.0238430)
Supplement: Attachment — Submitted filename: Response to Reviewers.docx [file pone.0238430.s004.docx]

The Social Network of Medical Case Managers, Housing Providers, and Health Department Staff in the Ryan White HIV/AIDS Program: A Midwest Case Study

| **Reviewer Comments** | **Authors’ response** | **Location in Revised Manuscript with Track Changes** |
| --- | --- | --- |
| Please ensure that your manuscript meets PLOS ONE's style requirements, including those for file naming. | We have reviewed the style requirements and made changes accordingly. | Main Text |
| Please ensure that you include a title page within your main document. We do appreciate that you have a title page document uploaded as a separate file, however, as per our author guidelines (<http://journals.plos.org/plosone/s/submission-guidelines#loc-title-page>) we do require this to be part of the manuscript file itself and not uploaded separately. Could you therefore please include the title page into the beginning of your manuscript file itself, listing all authors and affiliations. | We have included the title page in the main document. | Main Text, page 1 |
| Please provide an amended Funding Statement declaring this commercial affiliation, as well as a statement regarding the Role of Funders in your study. If the funding organization did not play a role in the study design, data collection and analysis, decision to publish, or preparation of the manuscript and only provided financial support in the form of authors' salaries and/or research materials, please review your statements relating to the author contributions, and ensure you have specifically and accurately indicated the role(s) that these authors had in your study. You can update author roles in the Author Contributions section of the online submission form. | We have included the following statement in our cover letter: “This project was funded by the U.S. Department of Health and Human Services, Health Resources and Services Administration, HIV/AIDS Bureau under prime award #U1SHA29299, subaward #9920160087. The funder did not provide any role in study design, data collection and analysis, decision to publish, or preparation of the manuscript. HRSA only provided funding for authors’ salaries and research materials.” | Cover Letter |
| Please also provide an updated Competing Interests Statement declaring this commercial affiliation along with any other relevant declarations relating to employment, consultancy, patents, products in development, or marketed products, etc. | Thank you for this comment. The RAND Corporation is a non-profit research organization and not a commercial company. Therefore, the steps outlined by the editor are not required. We have included the following statement in the cover letter: “The authors have no competing interests to declare. The RAND Corporation is a non-profit research organization and not a commercial company.” | Cover Letter |
| In your Data Availability statement, you have not specified where the minimal data set underlying the results described in your manuscript can be found. PLOS defines a study's minimal data set as the underlying data used to reach the conclusions drawn in the manuscript and any additional data required to replicate the reported study findings in their entirety. All PLOS journals require that the minimal data set be made fully available. For more information about our data policy, please see <http://journals.plos.org/plosone/s/data-availability>.  Upon re-submitting your revised manuscript, please upload your study’s minimal underlying data set as either Supporting Information files or to a stable, public repository and include the relevant URLs, DOIs, or accession numbers within your revised cover letter. For a list of acceptable repositories, please see <http://journals.plos.org/plosone/s/data-availability#loc-recommended-repositories>. Any potentially identifying patient information must be fully anonymized. | We have included deidentified data in the supplemental information files of this manuscript. | Supplemental Information and Main Text |
| The [PLOS Data policy](https://nam02.safelinks.protection.outlook.com/?url=http%3A%2F%2Fwww.plosone.org%2Fstatic%2Fpolicies.action%23sharing&data=01%7C01%7CTyler_Prochnow1%40baylor.edu%7Cf13a0738c9444f8bd8f408d82a03af26%7C22d2fb35256a459bbcf4dc23d42dc0a4%7C0&sdata=QcWrDFkeNobY%2Bv2NyEXu2NsZce7XwrNW3sGGviUxDEQ%3D&reserved=0) requires authors to make all data underlying the findings described in their manuscript fully available without restriction, with rare exception (please refer to the Data Availability Statement in the manuscript PDF file). The data should be provided as part of the manuscript or its supporting information, or deposited to a public repository. For example, in addition to summary statistics, the data points behind means, medians and variance measures should be available. If there are restrictions on publicly sharing data—e.g. participant privacy or use of data from a third party—those must be specified | We have created two files to satisfy this request: 1) a deidentified data set which includes all participants, demographics, and the network composition scores, and 2) an edge list which details all connections between the participants which was used for the ERGM analysis. | Supplemental Information |
| Please test the normality of reported network compositions. If they are not normal distributed, Kruskal–Wallis test (rank test) should be used. Median and interquartile range should be reported instead of mean and stand deviation. | We thank the reviewer for proposing this. We have conducted normality tests on the network composition scores, and several were not normally distributed. As such, we have shifted to Kruskal-Wallis tests where appropriate and updated the methods, results, and table reporting as appropriate. The application of KW tests did not affect overall findings in any meaningful way. | Main Text, page 7, lines 135-144 |
| In the ERGM analysis, race/ethnicity should be added as a model covariate. | Thank you for this suggestion. We have added race/ethnicity as a covariate in our models. | Main Text, page 9-14 |
| Your data and results are unique to the city and/or state represented. This is implied early in the paper but needs to be addressed throughout. Thus, you must change this statement in the beginning of the discussion (pg 10, lines 157-8) to read, "This study aimed to show the role of health department staff in care coordination for HIV treatment in a Midwest city." And change the following sentence to read, "We assessed a single Ryan White funded area..." I am confident you would have different results if data were collected from my state which also has a state health dept serving as a hub for HIV programs. | As suggested by the reviewer, we have included the proposed changes to contextualize our findings in the local setting. | Main Text, page 15, line 194 |
| The author should provide the number of invitations sent out for each role and whether the response rates varied by roles. This information is critical to test whether selection bias existed in this study. If the author had demographic characteristics of all invited individual. It will strengthen this study by exploring associations between demographics and response to survey. | We have included information on number of invitations and response rates in the main text. Five medical case managers did not respond to the survey. We do not have demographic data on the five individuals who did not respond, as all information was provided by the participants. Medical case managers accounted for 77% of the total sample of participants. | Main Text, page 5, lines 106-110; 113. |
| Please include additional information regarding the survey or questionnaire used in the study and ensure that you have provided sufficient details that others could replicate the analyses. For instance, if you developed a questionnaire as part of this study and it is not under a copyright more restrictive than CC-BY, please include a copy, in both the original language and English, as Supporting Information. In addition, please describe the development and validation of this tool in further detail, for example by referring to previous works and any pre-testing that took place. | We have now included the survey in the supplemental information and described the survey in more detail in the text. | Supplemental information and Main Text, page 5, lines 106-110. |
| Please refer to any post-hoc corrections to correct for multiple comparisons during your statistical analyses. If these were not performed please justify the reasons. Please refer to our statistical reporting guidelines for assistance ([https://journals.plos.org/plosone/s/submission-guidelines.#loc-statistical-reporting](https://nam02.safelinks.protection.outlook.com/?url=https%3A%2F%2Fjournals.plos.org%2Fplosone%2Fs%2Fsubmission-guidelines.%23loc-statistical-reporting&data=01%7C01%7CTyler_Prochnow1%40baylor.edu%7Ccf90adc4b47848bc273108d82e6e0315%7C22d2fb35256a459bbcf4dc23d42dc0a4%7C0&sdata=O6w%2Bgb8mZbGVAH8cOjVQfdOSO4XJRgq%2FxF4QvqGaE6Y%3D&reserved=0)). | Post-hoc corrections were not performed in this study due to the exploratory nature of this manuscript and analysis, as well as the relatively small sample size which was restricted by the network size. We have clarified this in the Discussion section as a limitation of the manuscript. | Main Text, Page 16, lines 231-233. |
